# Role of the paramagnetic donor-like defects in the high n-type conductivity of the hydrogenated ZnO microparticles

**DOI:** 10.1038/s41598-020-74449-3

**Published:** 2020-10-15

**Authors:** Dariya Savchenko, Andrii Vasin, Oleksandr Kuz, Igor Verovsky, Andrey Prokhorov, Alexey Nazarov, Jan Lančok, Ekaterina Kalabukhova

**Affiliations:** 1 Institute of Physics CAS, Prague, 182 21 Czech Republic; 2 National Technical University of Ukraine “Igor Sikorsky Kyiv Polytechnic Institute”, Kyiv, 03056 Ukraine; 3 V.E. Lashkaryov Institute of Semiconductor Physics, NAS of Ukraine, Kyiv, 03028 Ukraine

**Keywords:** Semiconductors, Magnetic properties and materials, Semiconductors, Electronic properties and materials, Magnetic properties and materials, Electronic properties and materials

## Abstract

The magnetic and electronic properties of the hydrogenated highly conductive zinc oxide (ZnO) microparticles were investigated by electron paramagnetic resonance (EPR) and contactless microwave (MW) conductivity techniques in the wide temperature range. The EPR spectra simulation allowed us to resolve four overlapping EPR signals in ZnO microparticles. The Lorentzian EPR line with isotropic *g*-factor 1.9623(5) was related to the singly ionized oxygen vacancy. Another Lorentzian line with *g*_||_ = 1.9581(5), *g*_⊥_ = 1.9562(5) was attributed to the zinc interstitial shallow donor center, while EPR signal with *g*_||_ = 1.9567(5), *g*_⊥_ = 1.9556(5) and Gaussian lineshape was assigned to the hydrogen interstitial shallow effective-mass-like donor. The EPR signal with *g*_||_ = 1.9538(5), *g*_⊥_ = 1.9556(5) and Lorentzian lineshape was tentatively attributed to the shallow donor center. The charge transport properties in ZnO microparticles have been investigated by the contactless MW conductivity technique at *T* = 5–296 K. Two conduction mechanisms, including ionization of electrons from the shallow donors to the conduction band and hopping conduction process, have been distinguished. The hopping conduction process follows Mott’s variable-range hopping *T*^−1/4^ law at *T* = 10–100 K. The evaluated values of the average hopping distance (15.86 Å), and hopping energy (1.822 meV at 40 K) enable us to estimate the donor concentration in the investigated ZnO microparticles as ~ 10^18^ cm^−3^.

## Introduction

Zinc oxide (ZnO) is a wide bandgap (3.37 eV) semiconductor material with excellent electrical and optical characteristics such as high n-type conductivity and optical transparency in the visible region. These properties make ZnO the promising material for the production of the optoelectronic devices, ultraviolet and white light-emitting diodes, photodetectors as well as for the preparation of the transparent electrodes for the thin-film amorphous silicon solar cells and different display panels^1–3^.

ZnO commonly exhibits n-type conductivity and is considered as promising low-cost, transparent conductive oxide material capable of transporting charge carriers and visible photons perspective for application in photovoltaic devices^4^. However, despite numerous investigations, the nature of this conductivity is still controversial. Traditionally, the conductivity of n-type in semiconductor materials was assigned for the existence of the intrinsic defects or impurities. The information about the origin of the intrinsic and impurity defects presented in ZnO mostly comes from electron paramagnetic resonance (EPR) spectroscopy measurements. Nevertheless, the interpretation of the EPR data relied mostly on the *g*-factor value of the signals because no convincing hyperfine (hf) structure was detected in the EPR spectrum of the defects, mainly due to the low concentration of magnetic ^17^O and ^67^Zn isotopes.

One set of the commonly observed EPR signals with isotropic *g*-factor *g*_iso_ ~ 1.955–1.964 was attributed to the singly charged oxygen (O) vacancy (${\text{V}}_{O}^{ \cdot }$)^5–8^, which can be compared with the F-centers in alkali halides (single line without any hf interaction). At the same time, the high-field EPR measurements in ZnO nanoparticles have resolved a small axial *g*-anisotropy, which designates axial site symmetry for the ${\text{V}}_{O}^{ \cdot }$^9^.

On the other hand, some authors favored assigning the EPR lines with *g* ~ 1*.*96 to conduction electrons originated from the ionization of shallow donor states connected with the presence of the impurities^10–13^ or zinc (Zn) interstitial (Zn_i_) defects^14^. Therefore, despite a large amount of available data, the assignment of the EPR signals at *g* ~ 1.955–1.964 remains unclear and is still under debate.

The other set of the EPR signals of isotropic and axial symmetry with *g* ~ 1.99–2.00 was assigned with surface defects attributed to the Zn_i_, negatively charged Zn vacancies (${\text{V}}_{Zn}^{ - }$) and in some reports to the positively charged ${\text{V}}_{O}^{ \cdot }$ observed in electron-irradiated ZnO and under photoexcitation^15,16^.

However, according to the first-principles calculations, none of the intrinsic defects can be the reason for the high carrier concentration of n-type ZnO^17^. The high n-type conductivity may be caused by shallow donor impurities incorporated into ZnO crystals^18,19^. Considering that the formation energy of hydrogen (H) atoms is low (1–2.5 eV), it was suggested that H atoms might be responsible for the conductivity of n-type in the ZnO matrix. Moreover, the effect of H donors on the resistivity is more significant than the impact of such donor-like defects as O vacancies (V_O_), V_Zn_, and Zn_i_^20^. Recently, a strong effect of the H on the resistivity of thin films of ZnO deposited by radio frequency magnetron sputtering of ZnO micropowder target utilizing Ar/H_2_ or Ar/CH_4_ mixture as working gas was reported^21^. The electrical resistivity of the hydrogenated ZnO films was reduced by more than three orders at room temperature compared to those deposited in pure Ar^21^. The first-principles calculations have shown that H in ZnO residing the interstitial or O positions may act as a shallow donor and contribute to the n-type conductivity^22^.

The previous EPR studies also resulted in direct observation of H in single crystals of ZnO. The involvement of H in the EPR signal structure was found directly by the electron-nuclear double resonance (ENDOR) method^23^. At the same time, the nuclear magnetic resonance (NMR) spectra measured in ZnO ceramics with a high-spin concentration of paramagnetic centers with *g* ~ 1.96 did not show any ^1^H signals^24^.

As was shown in^25^, the H incorporation in ZnO films may induce metal–semiconductor transition and occurrence of the low temperature (25–80 K) Mott’s variable range hopping motion. Moreover, shallow defect levels with the activation energy ranging from 20 to 2 meV were found from Hall-effect measurements in H-incorporated ZnO thin films^23^.

Recently performed first-principles calculations showed that H doping could cause a metal–insulator transition in metal oxide semiconductors such as wurtzite ZnO^26^. In particular, it was found that the doping with H leads to the filling of the conduction band-edge states, and the degree of the filling depends on the H concentration. Hence, it was concluded that the H-doping might induce the metallic behavior of ZnO^21,26^.

However, despite numerous experimental and theoretical investigations of the H role in the charge transport in ZnO bulk crystals, ceramics, powders, and thin films, the correlation between the type of the paramagnetic species and electrical conductivity in hydrogenated ZnO was not presented in the literature. In this work, the hydrogenated ZnO microparticles were studied using EPR spectroscopy at *T* = 80–5 K and the contactless method of microwave (MW) conductivity measurement at *T* = 296–5 K. For the first time, the simultaneously observed four donor-like centers around *g* ~ 1.96 were detected in the EPR spectra and unambiguously identified from their temperature behavior. In addition, the correlation between the observed paramagnetic species and the charge transport process has been established.

## Materials and methods

The commercial ZnO microparticles with the tabulated average grain size of about 5 μm were annealed at *T*_ann_ = 1000 °C for 30 min in the flow of pure H_2_ at atmospheric pressure. To minimize contaminations, the air was evacuated from the furnace by a mechanical vacuum pump down to the pressure of about 10 Pa, followed by input of pure H_2_ up to atmospheric pressure. For comparison, the ZnO microparticles annealed at *T*_ann_ = 1000 °C for 30 min in the N_2_ flow at atmospheric pressure were investigated as well.

The EPR spectra were measured on Bruker ELEXSYS E580 spectrometer at X-band (*ν* ~ 9.4 GHz) in the wide temperature interval using the ER 4122 SHQE SuperX High-Q cavity with ER 4112HV helium-flow cryostat. The temperature dependence of EPR spectra was measured with the following parameters: microwave power level was 0.3768 mW, the modulation amplitude is 0.1 mT, modulation frequency was 100 kHz, conversion time was 120 ms, and the spectral resolution was set to 2048 points. As a reference sample, a Bruker strong pitch (*g* = 2.0028) was used. For the elimination of E’ center defects and undesirable paramagnetic species, we have used the synthetic quartz sample tube (*d* ~ 4 mm). To simulate the EPR spectra, the EasySpin 5.2.28 MATLAB toolbox was utilized^27^.

The temperature dependence of MW conductivity of the ZnO microparticles was obtained utilizing a contact-free technique. The principal idea of this technique is that free carriers absorb the electrical component of the MW field, resulting in the variation of the EPR cavity quality factor (*Q*-factor)^28–34^.

## Experimental results

### EPR spectra of ZnO microparticles in the wide temperature interval

**Figure 1:**
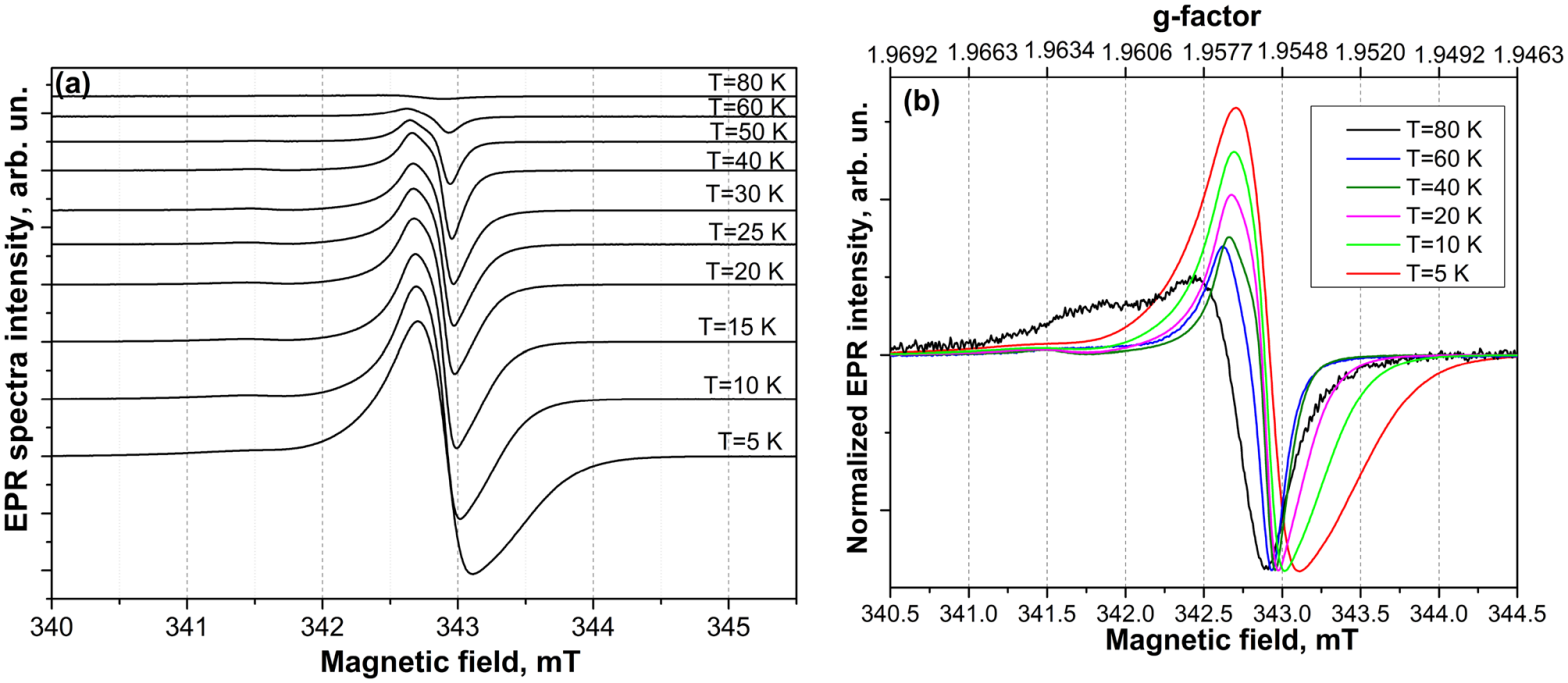
Temperature dependence of X-band EPR spectra (**a**) and EPR spectra with normalized to a minimum intensity value (**b**) measured in ZnO microparticles at temperatures from 80 to 5 K.

The EPR measurements were performed at temperatures from 80 to 5 K because at higher temperatures (*T* > 80 K), owing to the high conductivity of the samples, we were unable to critically couple the cavity, and consequently, no EPR signals were detected. Figure 1 demonstrates the temperature variation of the EPR spectra measured in ZnO microparticles. As it follows from Fig. 1a,b the EPR signal intensity increases with the temperature decrease and consists of several components with the *g*-values ranging between 1.95 and 1.97.

**Figure 2:**
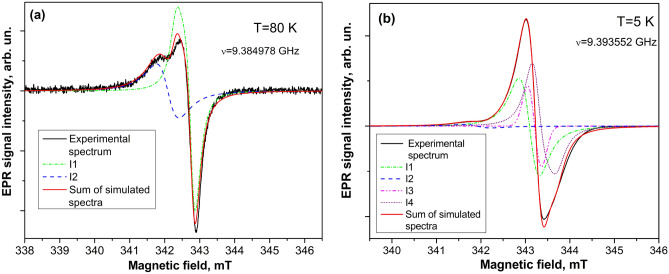
Experimental, simulated components and sum of simulated components of X-band EPR spectra measured in ZnO microparticles at 80 K (**a**) and 5 K (**b**).

Figure 2a,b presents the measured and simulated EPR spectra in ZnO microparticles at 80 K and 5 K. At *T* = 80 K, the EPR spectrum consists of two spectrally closed EPR signals with Lorentzian lineshape labeled as I1 and I2 of different intensity and linewidth. The I1 center was described by *g*_iso_ = 1.9623(5) and linewidth ~ 0.5 mT, while the I2 center of smaller intensity was characterized by *g*_||_ = 1.9581(5), *g*_⊥_ = 1.9562(5) and linewidth ~ 0.3 mT. With further lowering the temperature down to 60 K, two additional EPR signals labeled as I3 and I4 appeared in the EPR spectrum of ZnO microparticles. Figure 2b represents the EPR spectrum detected at 5 K, which consists of four overlapped components (I1, I2, I3, and I4). The I3 EPR signal described by *g*_||_ = 1.9567(5), *g*_⊥_ = 1.9556(5) has Gaussian lineshape and linewidth ~ 0.2 mT. The I4 EPR signal with *g*_||_ = 1.9538(5), *g*_⊥_ = 1.9556(5) has Lorentzian lineshape and linewidth ~ 0.1 mT.

**Figure 3:**
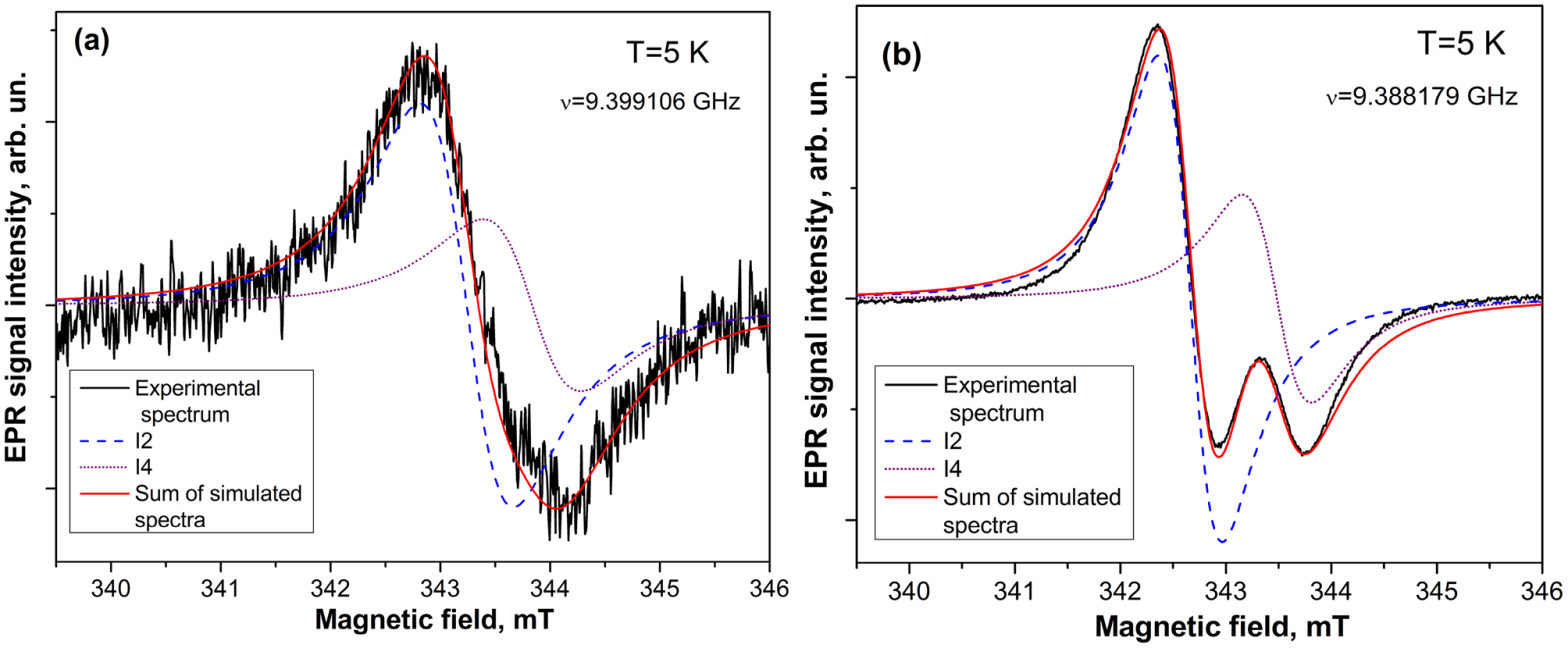
Experimental, simulated components and sum of simulated components of X-band EPR spectra measured at 5 K in the initial ZnO microparticles (**a**) and annealed ZnO microparticles in N_2_ atmosphere at 1000 °C (**b**).

In Fig. 3 the experimental and simulated EPR spectra measured at *T* = 5 K in the initial ZnO microparticles and in ZnO microparticles annealed in N_2_ atmosphere at 1000 °C are shown for comparison (the same sample volume was used as in the case of ZnO microparticles in H_2_ atmosphere). As it follows from Fig. 3, the EPR spectra consist of two broad (0.6–0.8 mT) components with average *g*-factor values 1.9570(5) and 1.9530(5) that correspond to I2 and I4 centers observed in ZnO microparticles annealed in the H_2_ atmosphere at 1000 °C, respectively. The intensity ratio of the paramagnetic centers observed at 5 K in the initial, annealed in N_2_ and H_2_ atmosphere ZnO microparticles was found to be: 0.003:0.06:1, showing that the spin concentration of paramagnetic centers significantly increases upon the annealing in H_2_ atmosphere.

The obtained parameters of the I3 and I2 EPR signals are similar to those found in ZnO single crystals, where two EPR lines were resolved at a MW frequency of 95 GHz with *g*_||_ = 1.9569(5), *g*_⊥_ = 1.9552(5) and *g*_||_ = 1.9571(5), *g*_⊥_ = 1.9555(5) and attributed to the donor centers D1 and D2, respectively^23^. The assignment of the D1 with a donor center in^23^ was based on the measurements of the ENDOR spectra. It was shown that the D1 center has hf coupling with over 50 shells of neighboring ^67^Zn nuclei, demonstrating that the wave function of the unpaired electron is delocalized, therefore D1 was attributed to the effective mass (EM) shallow donor. Besides, the observed ^1^H ENDOR signals from the D1 center indicates that this center should be related to the hydrogen (H) donor^23^. Later, the D1 and D2 centers were observed in ZnO powder in the temperature interval from 5 to 300 K, which was treated in the NH_3_ atmosphere, and attributed to H_i_ and Zn_i_^35^. The D2 center assignment with Zn_i_ in^35^ resulted from the ODMR investigation of the defects in irradiated ZnO crystals at low temperatures in^36^. Considering that the I3 EPR signal has Gaussian lineshape arising from unresolved hf interaction, we may also conclude that this interaction is caused by the presence of the ^1^H nuclei in the vicinity of the defect, which can cause the conductivity of n-type in ZnO^19^. Hence, it seems reasonable to attribute I3 and I2 EPR signals observed in ZnO microparticles to H_i_ and Zn_i_ centers, respectively.

To identify the I1 EPR signal, we have relied on the previous theoretical and experimental investigations of the donor-like defects in ZnO (V_O_ and Zn_i_), which gave us arguments to attribute it to ${\text{V}}_{O}^{ \cdot }$ generated by the reaction: V_O_ ↔ ${\text{V}}_{O}^{ \cdot }$ + e^−^. In the light of the latest first-principle calculations of the intrinsic defects in ZnO, the Zn_i_ is stabilized by the presence of the V_O_ and residual dopants such as H^37^. It was shown that the interaction between V_O_ and Zn_i_ gives rise to lowering their formation energy. Since Zn_i_ is a shallow-donor defect, the position of the Fermi level (*E*_F_) moves towards the conduction band (CB) minima, and the O-deficient ZnO shows n-type conductivity^38^. The theoretical predictions were confirmed experimentally by observing EPR signals from ionized ${\text{V}}_{O}^{ \cdot }$ and ${\text{Zn}}_{i}^{ + }$ at *g* ~ 1.959 and *g* ~ 1.955, respectively, in the EPR spectrum of ZnO nanoparticles in^7^.

The I4 EPR signal has the *g*-value similar to that observed early in ZnO crystals and attributed to the donor electrons, which move from one site to another by hopping conduction resulting in motional narrowing of single donor EPR signal^39^. Taking into account a small linewidth of the I4 signal, we may also tentatively attribute it to the donor center with the motional averaging effect.

Thus, unlike to the ZnO microparticles annealed in the H_2_ atmosphere, only two EPR signals of small intensity from Zni (I2) and shallow donor center (I4) were observed in initial and annealed in N_2_ atmosphere ZnO microparticles.

**Figure 4:**
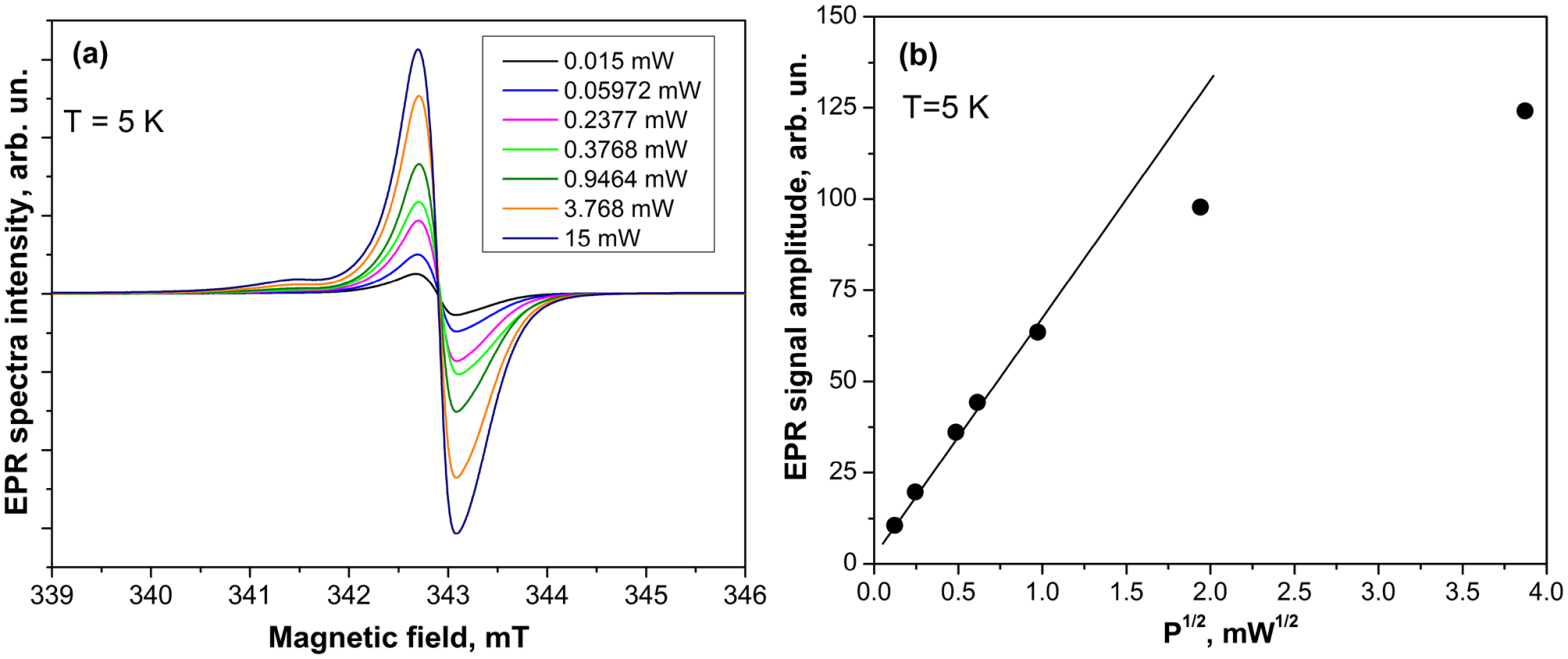
EPR spectra measured at X-band in ZnO microparticles depending on MW power (0.015–15 mW) (**a**). The plot of the peak-to-peak amplitude of the EPR signal dominated by shallow donors versus the square root of MW power (*P*^1/2^) (**b**).

On the other hand, in contrast to the bulk material, the core–shell model cannot be excluded when describing the defects in microsized ZnO samples. According to the model presented in^40^, the EPR signal at *g* ~ 1.961 was related to the core defect in ZnO nanoparticles. Besides, it was reported that the EPR signal assigned to the core defect does not show the saturation effect upon MW power variation^40^. Figure 4a shows the EPR spectrum in ZnO microparticles, which is a superposition of the four components measured with the different MW power levels varied in the range from 0.015 to 15 mW. As can be seen from Fig. 4b, the four-component EPR spectrum observed in ZnO microparticles shows the saturation behavior indicating that these spectra should not be associated with the core defects.

Analyzing the temperature variation of the EPR signal intensity of the observed paramagnetic centers, we have concluded that they do not follow Curie law. According to the Anderson model, the lack of the Curie-law paramagnetism for shallow donor centers can be explained by mixing of the localized levels with conduction states^41^.

### Temperature variation of MW conductivity in ZnO microparticles

**Figure 5:**
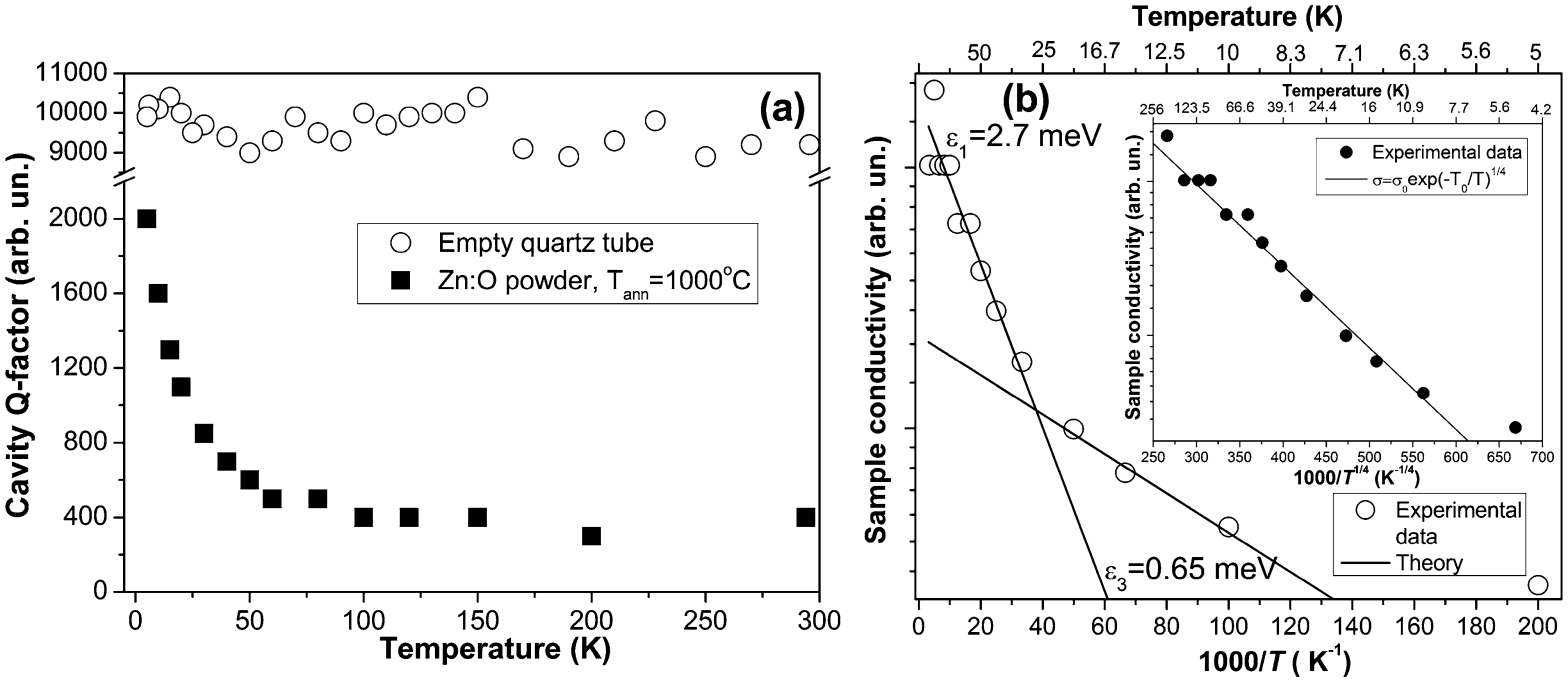
The temperature dependence of the *Q*-factor when the cavity was loaded with ZnO microparticles and with the empty quartz sample tube (**a**). The temperature variation of the logarithm of MW conductivity estimated from the cavity *Q*-factor for ZnO microparticles (**b**). The inset in (**b**) shows the ln(*σ*_MW_) versus *T*^−1/4^. *T* = 296–5 K.

The electrical conductivity of ZnO microparticles was investigated utilizing a contactless MW conductivity technique over the temperature range from 296 to 5 K. Figure 5a shows the variation of the *Q*-factor when EPR cavity was loaded with ZnO microparticles. It is well known that sample conductivity is inversely proportional to the cavity *Q*-factor; therefore, the observed increase of the cavity *Q*-factor with the temperature decrease is related to the reduction of the conductivity in ZnO microparticles with the temperature. From Fig. 5a, it can be seen that in the case when the cavity is loaded with the empty sample quartz tube, the *Q*-factor remains high and constant in the entire temperature range.

At the same time, comparing the *Q*-factor values of the cavity loaded with different ZnO microparticles at 295 K, we have concluded that the highest conductivity has the sample annealed in H_2_ atmosphere (*Q* = 400), a slightly lower conductivity shows the sample annealed in N_2_ atmosphere (*Q* = 1300), while the initial sample reveals very low conductivity (*Q* = 8000). A significantly higher conductivity in hydrogenated ZnO microparticles than that in the initial sample can be explained by the coexistence of ${\text{V}}_{O}^{ \cdot }$, Zn_i_, and H donors in hydrogenated ZnO.

Figure 5b demonstrates the temperature variation of the logarithm of MW conductivity (that is proportional to 1/*Q*) for ZnO microparticles that can be theoretically described as:

1 $$\sigma_{{{\text{MW}}}} (T) = \sigma_{1} \exp \left( { - \varepsilon_{1} /kT} \right) + \sigma_{3} \exp \left( { - \varepsilon_{3} /kT} \right),$$

where *ε*_1_ is the activation energy of the electrons from the donor state to the CB, *ε*_3_ is the activation energy of electron jumping between occupied and unoccupied donor states (hopping conduction process).

From the fitting of Eq. (1) with experimental data shown in Fig. 5b, we have estimated *ε*_1_ = 2.7 meV and *ε*_3_ = 0.65 meV. The obtained low value of the electron thermal ionization energy indicates the high carrier concentration in ZnO microparticles and the formation of the shallow impurity band. The obtained value is consistent with the thermal activation energies ranging between 1.5 and20 meV for H-related donors obtained from Hall effect measurements in hydrogenated ZnO:H films^25^. The conductivity with the activation energy *ε*_*3*_ can be ascribed to the hopping conduction process, taking place in the shallow impurity band at *T* < 25 K.

According to the Mott’s variable range hopping model, the variation of the conductivity with the temperature can be described by the expression^42,43^:

2 $$\sigma (T) = \sigma_{0} \exp \left( { - T_{0} /T} \right)^{1/d + 1} ,$$

where *σ*_0_ is the temperature-independent constant, $T_{0} = 18/(kN(E_{F} )\xi^{3} )$ is the specific temperature that depends on the localization length of the relevant electronic wave function (ξ) and the electronic density of states at *E*_F_ (*N*(*E*_F_)) without electron–electron interactions, *d* is the dimensionality.

The fitting of the Eq. (2) with experimental data presented in the inset in Fig. 5b yields *d* = 3. Therefore, we have ascertained that the investigated ZnO microparticles represent a 3D system*.*

According to^44^, the average hopping distance ($\overline{R}_{hop,Mott}$) and the average hopping energy ($\overline{W}_{hop,Mott}$) of the conduction electrons can be written as:

3 $$\overline{R}_{hop,Mott} = \left( {\frac{3}{8}\xi } \right)\left( {\frac{{T_{0} }}{T}} \right)^{1/4} \approx \left( {\frac{3}{8}a_{B} } \right)\left( {\frac{{T_{0} }}{T}} \right)^{1/4} \;{\text{and}}\;\overline{W}_{hop,Mott} = \left( \frac{kT}{4} \right)\left( {\frac{{T_{0} }}{T}} \right)^{1/4} .$$

From the fitting of Eq. (2) with experimental data, we have obtained that *T*_0_ = 800 K. If we take the electronic localization length as the effective Bohr radius for such shallow donors as V_O_, Zn_i_, and H impurity atoms in ZnO (*a*_B _≈ 2 nm)^45^, we can calculate that: *N*(*E*_F_) = 2.037 × 10^47^ J^−1^ m^−3^, $\overline{R}_{hop,Mott}$ = 15.86 Å, $\overline{W}_{hop,Mott} (40{\text{K}} )$ = 1.822 meV. The obtained values are close to those obtained in^46^ for ZnO polycrystalline films with the resistivity of about 2 Ω cm at 300 K and donor concentration as high as 10^18^ cm^−3^.

According to^45^, a high donor concentration leads to the reduction of the thermal activation energy involved in the variable range hopping conduction process resulting in a lower value of $\overline{W}_{hop,Mott}$. The criterion ${{\overline{R}_{hop,Mott} } \mathord{\left/ {\vphantom {{\overline{R}_{hop,Mott} } {\xi > 1}}} \right. \kern-\nulldelimiterspace} {\xi > 1}}$ was not satisfied in our ZnO microparticles because the samples have sufficiently high conductivity to be far enough from the insulating side of the metal to insulator phase transition^45^. On the other hand, in accordance with^42,47^, the metal to insulator transition in ZnO single crystals appears at a donor concentration of ~ 5 × 10^18^ cm^−3^ above that variable range hopping conduction process does not affect the charge transport process.

We should note that at *T* < 20 K, the transition from the Mott type (1/*T*)^1/4^ to the Efros and Shklovskii type (1/*T*)^1/2^ of the variable range hopping conduction was observed early in polycrystalline ZnO films. However, it was found that the transition from the Mott to Efros and Shklovskii type of the variable range hopping conduction occurs in the samples with donor concentration lower than 1 × 10^18^ cm^−3^ and the value *N*(*E*_F_) lower than 1.3 × 10^47^ J^−1^ m^−3^ when long-range Coulomb interactions should be taken into account^48^.

Considering that the hopping conduction process occurred between occupied and unoccupied nearest-neighbor states in the impurity band, the availability of a sufficient number of empty ionized donor sites is required, which can be achieved at low temperatures by compensation of the shallow donors by deep defects. In this regard, the ${\text{V}}_{O}^{ \cdot }$, which behaves as a deeper donor than EM shallow ones, may be considered as compensating levels in ZnO microparticles leading to the creation of a partially filled donor impurity band.

## Discussion

Shallow donors related to the charge transport process that occurred in hydrogenated ZnO microparticles have been studied by EPR spectroscopy*.* The EPR spectra were measured over the temperature range from 5 to 80 K. From the temperature behavior of the EPR spectra measured in ZnO microparticles, four overlapping EPR signals have been resolved and assigned to the donor-like defects.

Two of them I2 [*g*_||_ = 1.9567(5), *g*_⊥_ = 1.9556(5)) and I3 (*g*_||_ = 1.9581(5), *g*_⊥_ = 1.9562(5)] have *g*-values similar to those found previously for D2 and D1 shallow donor centers reported in single crystals of ZnO^23^ and ZnO powders treated in the NH_3_ atmosphere^35^, which were assigned with Zn_i_ shallow donor and H_i_ shallow EM-like-donor centers, respectively. Considering that I2 EPR signals appeared in the EPR spectrum at temperature higher (at 80 K) than I3 EPR signals (at 60 K) the energy level of the Zn_i_ shallow donor in the bandgap should be slightly deeper than the H_i_ EM-like-donor.

The I1 EPR signal observed at *g*_iso_ = 1.9623(5) with linewidth ~ 0.5 mT was assigned to ${\text{V}}_{O}^{ \cdot }$. The main argument for the assignment of the I1 signal with ${\text{V}}_{O}^{ \cdot }$ was the theoretical prediction that Zn_i_ is stabilized in n-type ZnO by the presence of the high concentration of the oxygen vacancies V_O_^37^. This assignment is supported by the fact that Zn_i_ and ${\text{V}}_{O}^{ \cdot }$ centers simultaneously appeared in the EPR spectra of ZnO micropowders at *T* = 80 K, while EPR signals from H_i_ and shallow donor centers appeared at *T* < 60 K. It means that Zn_i_ and ${\text{V}}_{O}^{ \cdot }$ centers should have deeper energy levels than H_i_ that is in agreement with the literature data and supports our assignment of the four overlapping EPR signals.

Taking into account that I4 EPR signal appeared in the EPR spectrum along with the I3 signal, which was identified as H_i_ shallow EM-like-donor, we tentatively attributed the I4 signal to the donor with a motional averaging effect due to its small linewidth.

The spin Hamiltonian parameters of paramagnetic centers observed in hydrogenated ZnO microparticles and literature data are listed in Table 1.

**Table 1:** The spin Hamiltonian parameters of paramagnetic centers with *S* = 1/2 observed in hydrogenated ZnO microparticles along with literature data.

Center	*g*_||_	*g*_⊥_	*g*_av_^a^	References
I1 (${\text{V}}_{O}^{ \cdot }$)	1.9623(5)	1.9623(5)	1.9623(5)	This work
${\text{V}}_{O}^{ \cdot }$	1.96	1.96	1.96	^7,49–51^
I2 (Zn_i_)	1.9581(5)	1.9562(5)	1.9568(5)	This work
D2 (Zn_i_)	1.9571(5)	1.9555(5)	1.9560(5)	^23^
I3 (H_i_)	1.9567(5)	1.9556(5)	1.9559(5)	This work
D1 (H_i_)	1.9569(5)	1.9552(5)	1.9558(5)	^23^
I4 (shallow donor)	1.9538(5)	1.9556(5)	1.9550(5)	This work
Shallow donor	1.953	1.955	1.954	^39^

^a^Average *g*-value: *g*_av_ = (2*g*_⊥_ + *g*_||_)/3.

The charge transport properties in ZnO microparticles have been investigated by the contactless MW conductivity method. Two conduction mechanisms, including ionization of electrons from the donor energy levels and hopping conduction process, have been distinguished in the temperature range 5–300 K. From the theoretical analysis of the experimental data, two energy activation values *ε*_1_ = 2.7 meV and *ε*_3_ = 0.65 meV were obtained. The ionization energy value *ε*_1_ is consistent with the thermal activation energy for H donors obtained from Hall effect measurements in ZnO:H films ranging between 1.5 and 20 meV. It was found that the increase of the H doping in ZnO:H films leads to the decrease of the activation energy of the donor states due to the formation of the impurity band under the CB edge^25^. In comparison with ZnO:H films, the temperature-dependent Hall measurements in ZnO single crystals with a carrier concentration of about 6 × 10^16^ cm^−3^ yield activation energies for two donor states *E*_*D*1_ = (35 ± 5) meV and *E*_*D*2_ = (66 ± 5) meV^23^.

The obtained value of the *ε*_3_ = 0.65 meV coincides with the activation energies for the variable range hopping conduction found in ZnO thin films to be below 1 meV^52,53^, and thus corresponds to the activation energy of the Mott’s variable range hopping following *T*^−1/4^ law in the low-temperature range 90–10 K. The evaluated values of the average hopping distance $\overline{R}_{hop,Mott}$ = 15.86 Å, and hopping energy $\overline{W}_{hop,Mott} (40{\text{K}} )$ = 1.822 meV are found to be close to those obtained in ZnO polycrystalline films with a donor concentration of about 10^18^ cm^−3^ that corresponds to the critical concentration for the metal–insulator transition in ZnO (~ 5 × 10^18^ cm^−3^). Considering that the variable range hopping process occurs between an occupied (below the *E*_F_ position) and an unoccupied nearest neighbor state (above the *E*_F_ position), the existence of the ionized donor states is required. The presence of the V_O_ may lead to the appearance of a partially filled donor impurity band, and as a result, the Mott’s variable range hopping conduction mechanism takes place. The study of the electrical conduction process in ZnO thin films has supported this assumption. As was shown in^52^, the ZnO films deposited in O-deficient conditions exhibit a large concentration of donors, and the shallow donor impurity band was formed in these films in contrast to the ZnO single crystals. A large amount of V_O_ leads to a decrease in the resistivity of the sample. Thus, the relation between shallow donor centers and the charge transport process in hydrogenated ZnO microparticles was established.

## Conclusions

We have investigated the magnetic and electronic properties of the hydrogenated highly conductive ZnO microparticles by EPR and contactless MW conductivity methods. It was found that four paramagnetic centers are responsible for the conduction process that occurred in ZnO microparticles in the temperature range from 5 to 80 K. Two of them, including hydrogen (H_i_) and zinc (Zn_i_) interstitials, acting as shallow EM-like-donor and shallow donor centers, respectively, are responsible for the high conductivity of the hydrogenated ZnO microparticles. The analysis of the temperature variation of the MW conductivity allowed us to obtain the ionization energy of the electrons from H_i_ and Zn_i_ energy levels to the CB that was found to be *ε*_1_ = 2.7 meV. A small value of the ionization energy *ε*_1_ indicates the high carrier concentration in the investigated ZnO microparticles and the appearance of the impurity band below the CB edge. The third paramagnetic center was attributed to the singly ionized O vacancy (${\text{V}}_{O}^{ \cdot }$), which is a deep donor center. It was found that the Mott variable range hopping conduction process controls the charge transport characteristics of the ZnO microparticles in the temperature interval from 10 to 100 K with the activation energy of *ε*_3_ = 0.65 meV. Considering that the variable range hopping process occurs between an occupied state and an unoccupied nearest neighboring state, the ${\text{V}}_{O}^{ \cdot }$ can be considered as a compensating center. The fourth EPR signal was tentatively attributed to the shallow donor with a motional averaging effect.
